# Slipped capital femoral epiphysis: a population-based study

**DOI:** 10.1186/s12891-017-1665-3

**Published:** 2017-07-18

**Authors:** Bengt Herngren, Margaretha Stenmarker, Ludek Vavruch, Gunnar Hagglund

**Affiliations:** 1 Department of Clinical Sciences Lund, Orthopaedics Department of Orthopaedics, Ryhov county hospital, Lund University, SE-551 85 Jonkoping, Sweden; 2 Futurum Academy for Health and Care Jonkoping County Council, Department of Paediatrics, Ryhov county hospital, SE-551 85 Jonkoping, Sweden; 3 Faculty of Medicine and Health Sciences, Linkoping university, SE-581 83 Linkoping, Sweden; 4 Department of Clinical Sciences, Lund, Orthopaedics, Lund University, Box 117, SE-221 00 Lund, Sweden

**Keywords:** Epidemiology, Slipped capital femoral epiphysis, Incidence, Hip, Slipped upper femoral epiphysis

## Abstract

**Background:**

Slipped capital femoral epiphysis (SCFE) is the most common hip disorder in children 9–15 years old. This is a population-based study in Sweden presenting the epidemiology for SCFE.

**Methods:**

In a prospective cohort study, we analysed pre- and postoperative radiographs, medical records for all children treated for SCFE in Sweden 2007–2013, demographic data, severity of slip and surgical procedures performed.

**Results:**

We identified 379 Swedish children with primary SCFE 2007–2013; 162 girls, median age 11.7 (7.2–15.4) years, and 217 boys, median age 13 (3.8–17.7) years. The average annual incidence was 4.4/10000 for girls and 5.7/10000 for boys 9–15 years old. Obesity or overweight was found in 56% of the girls and in 76% of the boys. As an initial symptom, 66% of the children had hip/groin pain and 12% knee pain. At first presentation, 7% of the children had bilateral SCFE. Prophylactic fixation was performed in 43%. Of the remaining children, 21% later developed a contralateral slip.

Fixation with implants permitting further growth was used in >90% of the children. Femoral neck osteotomy was performed for 11 hips.

**Conclusions:**

The annual average incidence 2007–2013 in Sweden showed a mild increase for girls. The male-to-female ratio was lower than previous regional data from Sweden. Overweight or obesity is one major characteristic for boys with SCFE but to a less extent for girls. Knee pain as initial symptom cause a delay in diagnosis. Most hospitals in Sweden treat <2 children annually.

## Background

Slipped capital femoral epiphysis (SCFE) is the most common hip disorder in children 9–15 years old [1, 2]. The aetiology appears to be multifactorial. Identified biomechanical factors are obesity, increased femoral retroversion and increased physeal obliquity that all result in increased shear forces in the capital physis [3–5]. SCFE is known to be associated with endocrine disorders, e.g. hypothyroidism, hypogonadism, and hypopituitarism [6–8]. Children with renal failure osteodystrophy [9] or children who have acquired radiation therapy to the pelvis [10, 11] are also known to have a higher risk to develop SCFE.

SCFE is a separation between the epiphysis and the metaphysis of the proximal femur. The epiphysis remains in the acetabulum while the femur usually rotates outwards and in extension [12, 13]. The duration of symptoms is correlated with increased severity of the slip [14–20]. A more severe slip is associated with increased pain and a further limitation in the range of motion [18, 21, 22]. Epidemiological data for SCFE from different countries have been presented, but only a few are based on a total national population [2, 23–26]. The incidence reported is related to ethnicity and sex. A seasonal variation has also been described but with uncertainty concerning the role in the ethiology of SCFE [2, 27–32]. A male-to-female ratio between 1.1:1 and up to 4.1:1 has been reported [1, 2, 23, 25, 28, 33, 34]. Previous reports with a total national population are based on data from national registers, e.g. Kids’ Inpatient database in the USA [2] or the national hospital registration system of The Netherlands [23].

The aim of this study was to describe the epidemiology for SCFE in Sweden and to identify the demographic characteristics of children, type and degree of SCFE, and the surgical procedures performed.

We hypothesized that: (1) there is a difference in incidence between boys and girls, (2) knee pain as presenting symptom will cause a difference compared to hip/groin pain when you compare to what extent an acute hip disorder would be suspected at the first health care contact, (3) age at diagnosis has not changed compared to previous regional epidemiological data from Sweden, and (4) overweight or obesity are predominating characteristics for children with SCFE in Sweden.

## Methods

This is a prospective cohort study of the total population of children treated for SCFE of the index hip in Sweden 2007–2013. All children were consecutively reported to a national quality register with one of the authors (BH) as director.

Inclusion criteria were: children living in Sweden, registered in the Swedish Population Register with a Swedish personal identity number, with SCFE in the index hip during the study period. Exclusion criteria were: SCFE because of high energy trauma or septic coxitis.

All children treated for SCFE were admitted to hospital for primary treatment. For control purpose of the completeness achieved in this study we were retrospectively provided with individual-based data from the Swedish National Board of Health and Welfare, and compared our database with the Swedish National Patient Register. All hospitals are directed by Swedish regulations to register all admissions with codes for diagnosis (WHO classification ICD-10) and treatment codes according to the NOMESCO - NCSP classification of surgical procedures (NOMESCO - Nordic Medico-Statistical Committee, NCSP - Nordic classification of surgical procedures). All hospitals that treated SCFE in Sweden participated.

Population data was collected through official statistics for Sweden (Statistics Sweden). The number of children 9–15 years old with a Swedish personal identity number was slightly higher for both girls and boys at the beginning of the study period, see Table 1. For that reason, we chose to use the average population of children 9–15 years old as the average population at risk when calculating the average annual incidence for SCFE during the study period. We excluded the five girls <9 years old and the boy of 3.8 years together with the four boys ≥16 years old when calculating the average annual incidence.

**Table 1:** Number of girls and boys, 9–15 years old, living in Sweden in 2007–2013

Year	2007	2008	2009	2010	2011	2012	2013	2007–2013 average
Girls	386,372	371,151	357,163	346,664	337,959	336,135	339,852	353,614
Boys	406,293	390,426	375,523	365,425	357,094	354,890	359,177	372,690
Total	792,665	761,577	732,686	712,089	695,053	691,025	699,029	726,304

Medical records together with pre- and postoperative radiographs were obtained for all patients and were analysed consecutively. Missing data were completed by contact with the hospital involved or the family. A follow-up was made after 24 months through an assigned contact person in each hospital to identify any additional surgery performed for SCFE on the contralateral hip.

From the medical records, we obtained information about: sex, place of residence, type of initial symptoms, initial health care provider, duration of symptoms, age at diagnosis, whether the SCFE was acute or not, type of surgical treatment, reduction manoeuvres applied [35–38], implant used and whether prophylactic surgery was performed on the contralateral hip. For osteotomies, we recorded whether a capital realignment procedure [39–41] was performed, with or without surgical dislocation of the hip [40]. Other specific surgical treatment methods registered were open reduction and internal fixation according to Parsch [42] and whether joint aspiration [43, 44] was performed for unstable SCFE.

The medical records revealed that height and weight were not routinely measured in the paediatric departments and therefore not reported to us. We then retrospectively asked the families for an additional informed consent to be signed to be able to obtain the growth curves from the school health nurse. We accepted a value for age adjusted BMI according to Karlberg 2001 [45] within 12 months before or after the primary surgery was performed for the index hip.

For both the analysis of the duration of symptoms and initial health care provider, we used the history that was presented on hospital admission for primary surgery for SCFE. The total duration of symptoms, i.e. time from initial presenting symptom until primary surgery was performed, was calculated in months. Surgery performed on the first day with symptoms was equal to 0 months; ≥1 day, but ≤1 week was equal to 0.25 months; >1 week, but ≤2 weeks was equal to 0.50 months; >2 weeks, but ≤3 weeks was equal to 0.75 months and so forth.

For the analysis of seasonal variation we excluded the children with >24 months duration of symptoms [30].

In children with bilateral SCFE at primary admission the index hip was the one with the longest duration of symptoms. When the duration of symptoms was equal for both hips, we designated the hip with the largest slip angle to be the hip with primary SCFE. For bilateral cases, only the data for the index hip were used except when comparing the parameters for the first and second slipped capital femoral epiphysis [1].

**Fig. 1:**
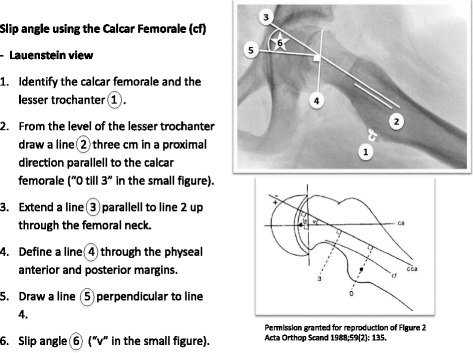
The slip angle measured according to the calcar femorale method (Hansson et al.)^1^. ^1^Permission has been obtained to use the figure from Hansson et al. 1988

**Fig. 2:**
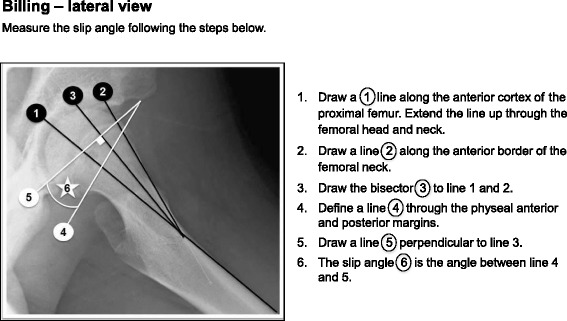
The slip angle measured according to the Billing method (Billing et al.)

The radiographic analysis included measurement of the slip angle on a Lauenstein view using the calcar femorale method [46] (Fig. 1). If a Billing lateral view was obtained the Billing method [47] was used (Fig. 2). For both these methods a minimum slip angle of 13^°^ was required for diagnosis [12, 46, 48]. If no lateral view was obtained, because of an unstable SCFE [49]*,* the Southwick head-shaft angle HSA [50] on the anteroposterior (AP) view was used. There is no cut off value described for SCFE in the literature using the HSA in the AP view. We, therefore, used the following criteria: HSA on an AP view of ≤110° together with a broken Klein’s line [51, 52] and clinical findings/symptoms of a hip disease, e.g. limping or groin/hip pain.

Hips with a slip angle of 13° to <30° were classified as mild, 30° to <50° as moderate, and ≥50° as severe [12, 17, 53, 54]. HSA of ≤110° on AP view was classified as severe.

A valgus SCFE was defined as a superior/lateral and posterior displacement of the proximal femoral epiphysis in relation to the femoral neck with an increased prominence of the lateral femoral epiphysis in relation to the lateral femoral neck (Klein’s line) on an anteroposterior view. For a unilateral valgus SCFE an increased HSA was also noted compared to the contralateral hip [55, 56].

The clinical classification described by Loder et al. [49] was used where the SCFE was either stable or unstable (not being able to ambulate with two crutches). Information from the medical records on admission to the hospital or from the description of the surgical procedure performed was used for the clinical classification.

All statistical analyses were performed using SPSS Statistics for Windows (version 22.0; IBM Corp, Armonk, NY). Continuous data were reported as the median with minimum and maximum values. Discrete data were reported as frequencies and/or percentages. For nonparametric tests between two independent groups we used a Mann-Whitney U-test. To compare proportions between two independent groups we used a cross-table and a chi-square test. A comparison of the Mann-Whitney U and chi-square tests was made using a Fischer exact test that found identical results.

## Results

### The annual average incidence

A total of 379 Swedish children with primary SCFE in 2007–2013 were identified. Of these, 35 were retrospectively identified through the Swedish National Patient Register. The average number of children 9–15 years old in Sweden in 2007–2013 was 726,304 (353,614 girls and 372,690 boys). Calculating the average annual incidence for children 9–15 years old we excluded the five girls and the one boy <9 years old together with the four boys ≥16 years old. The average annual incidence of SCFE was 4.4 per 10,000 girls and 5.7 per 10,000 boys 9–15 years old.

### Primary SCFE

**Fig. 3:**
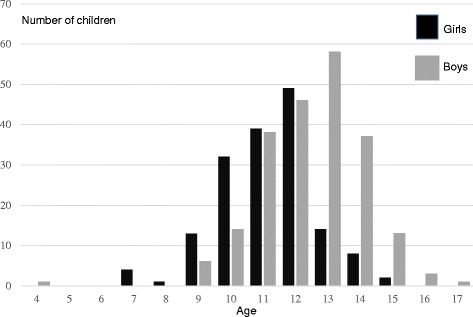
Age distribution

The median age at diagnosis for the 162 girls was 11.7 (7.2–15.4) with a mean of 11.6 years and for the 217 boys, it was 13.0 (3.8–17.7) with a mean of 12.9 years (Fig. 3). The youngest boy (3.8 years old) had a comorbidity of microcephaly together with cerebral palsy.

### Age-adjusted body mass index

We obtained data for 131 of the 162 girls and for 176 of the 217 boys i.e. for 81% of the cohort population. For the girls 74 of 131 (56%) and for the boys 133 of 176 (76%) were overweight or obese.

### Symptoms and duration (missing data, *n* = 4)

Most children had hip/groin pain as an initial symptom, but mixed symptoms and knee pain were also frequent. The median duration from onset of symptoms until diagnosis of SCFE was 2 months (0 to 48 months; missing data, *n* = 5). For the 44 children (15 girls and 29 boys) with knee pain as the main symptom, the median duration was 4 months (0.5 to 18 months) whereas for the 250 children with hip/groin pain as main symptom the median duration was 2 months (0.25 to 48 months). The difference in median duration of symptoms between knee and hip/groin pain as an initial symptom was statistically significant for the whole population (*p* = 0.004) but when the same analysis was made for boys and girls separately the difference was only statistically significant for boys (*p* = 0.004).

### Initial health care provider (missing data, *n* = 4)

Most children (250/379) were initially examined by their general practitioner. Eighty-five children were seen at the emergency care room in a hospital initially. The remaining children were primarily seen in an outpatient setting either by a paediatrician, a physician in school health or by a physiotherapist or a chiropractor/naprapath. For 247 of the 379 children (65%), a hip disorder was suspected with referral for a radiographic hip examination at first presentation to a health care provider. Of the 44 children with knee pain as their main symptom, 12 (27%) were sent for a radiographic hip examination at first consultation. Of the 250 children with hip or groin pain as their main symptom, 180 (72%) were sent for a radiographic hip examination at first consultation. There was a statistically significant difference between the proportion of children with knee pain that were sent for a radiographic hip examination compared with children with hip/groin pain as an initial symptom (*p* = 0.033).

### Duration of symptoms and slip severity

**Fig. 4:**
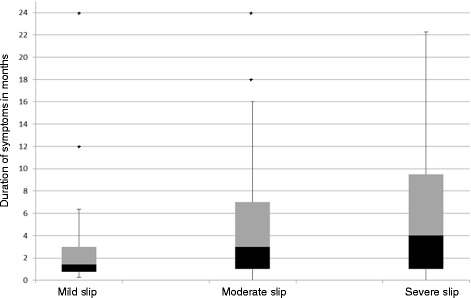
Severity of the slip in relation to duration of symptoms (missing data, *n* = 5)^1^. ^1^Outliers with duration of symptoms >24 months:. Mild (*n* = 1). Moderate (*n* = 1). Severe (*n* = 2)

The severity of the slip was associated with an increase in median duration of symptoms (Fig. 4).

Of the *89 severe slips*; 36 were in girls with a median age of 12 (9–15) years, 53 were in boys with a median age of 13 (10–16) years.

Of the *125 moderate slips*; 66 were in girls with a median age of 12 (8–14) years, 59 were in boys with a median age of 13 (9–16) years. Two slips were in valgus SCFE.

Of the *165 mild slips*: 60 were in girls with a median age of 11 (7–15) years, 105 were in boys with a median age of 12 (4–17) years. Two slips were in valgus SCFE.

### Seasonal variation

**Fig. 5:**
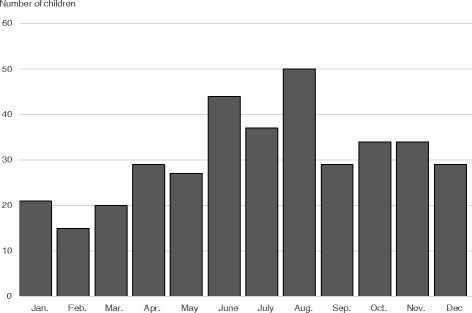
Seasonal variation for the study period 2007–2013 (missing data, *n* = 5)

The month of onset of symptoms was used to describe the seasonal variation of SCFE (Fig 5). Four children were excluded due to duration of symptom >24 months.

### Urban/rural location

One child had a protected residence and two children temporarily resided in an urban area but it was not possible to find any information for their ordinary place of domicile. For 64% of the children an urban area and for 35% a rural area was found for their place of domicile. Using the same definition of rural area, i.e. <5000 inhabitants, according to Statistics Sweden 26% of children (0–19 years old) lived in a rural area during the study period.

### Co-morbidity

In Table 2 the associated disorders are listed.

**Table 2:** Co-morbidity

Disorder	Number
Neurocognitive disorder (ADD, ADHD, Autism)	15
Thyroid insufficiency	2
Down’s syndrome	2
Other chromosome abnormalities	3
Hypopituitarism	1
Early onset of puberty	1
Late onset of puberty	1
Adrenoleukodystrophy	1
Short stature (treated with growth hormone)	1
Vitamin D deficiency	1
Diabetes mellitus	1
Osteopetrosis	1
Marfan’s syndrome	1
Cerebral palsy (with concomitant microcephaly)	1
Embryonal rhabdomyosarcoma (irradiation to pelvic area)	1
Fibrous dysplasia of proximal femur	1

### Age adjusted body mass index

Data was available for 81% of the cohort population concerning age-adjusted Body Mass Index. For the boys 76% and for the girls 56% were either obese or had overweight.

### Surgical procedure and implants

**Fig. 6:**
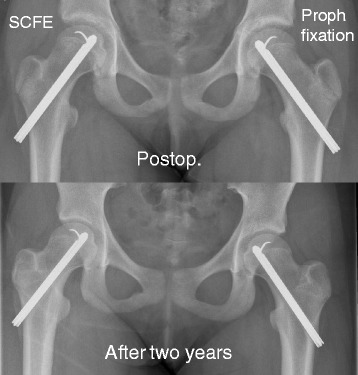
Implant for fixation that will allow for further growth of the femoral neck (Hansson pin)

The implants that were chosen and the surgical procedures performed are presented in Table 3. All mild and moderate slips were treated with a screw or pin fixation. Screws and pins were predominantly inserted using a percutaneous approach. Fixation with an implant permitting further growth of the femoral neck, e.g. Hansson hook pin (Fig. 6) or a screw with an extra short thread length, was used for >90% of the patients for both surgery for the index hip and when prophylactic pinning was performed. Twelve of these percutaneous procedures for the index hip had to be extended to a more invasive surgical approach because of difficulties in finding the correct entry point.

**Table 3:** Method of treatment related to severity of SCFE

Surgical treatment	Mild	Moderate	Severe	Total
Hansson hook pin	96	71	38	205
Cannulated screw with extra^a^ short thread length	58	43	32	133
Cannulated screw with short^b^ thread length	10	10	4	24
Multiple cannulated screws (diameter < 6 mm)	–	1	–	1
Multiple pins (diameter < 3 mm)	1	–	2	3
Capital realignment procedure with surgical dislocation of the hip	–	–	8	8
Capital realignment procedure without surgical dislocation of the hip	–	–	3	3
Open reduction and fixation without surgical dislocation of the hip (Parsch)	–	–	2	2
Total	165	125	89	379

^a^Specially designed screws with extra short thread length that will allow further growth of the femoral neck

^b^Ordinary short thread length i.e. approximately 16 mm

### Unstable SCFE

Sixty hips were unstable at surgery: 40 of these were severe, and 20 were mild or moderate slips (one of these was a valgus SCFE). Two of the 60 unstable SCFE had an open reduction with internal fixation, and three had a capital realignment procedure with a surgical dislocation performed, all with a severe SCFE.

Fifty-five of the 60 unstable slips were treated with internal fixation without open reduction.

For 31 of the 55 unstable slips treated without an open reduction, an intentional closed reduction manoeuvre was also performed. Nine of these had a mild or moderate slip, whereas 22 had a severe slip.

For 7 of the 55 unstable SCFE treated without an open reduction a joint aspiration was performed.

### The contralateral hip

**Fig. 7:**
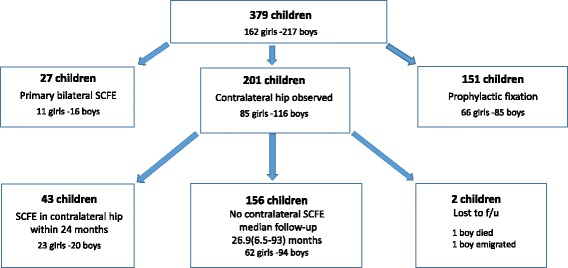
Study population

The study population is described in Fig. 7. A total of 27 children had bilateral SCFE at initial presentation. Prophylactic fixation was performed in 151 of the remaining 352 children (43%). For the 43 children with a later slip in the contralateral hip we observed a mean interval of 10.8 months between surgery for the index and the contralateral hip. Apart from an outlier with 59.8 months’ interval, the second largest interval was 24 months. The child with only a 0.5-month interval had no symptoms but had a lateral view radiograph analysed during the first admission. This child was then re-admitted only after 2 weeks, now with symptoms from the contralateral hip and with a contralateral SCFE diagnosed. Twelve of the 43 contralateral slips were diagnosed more than 1 year after the index slip.

For the 156 children with no contralateral hip affection during the follow-up we had a mean follow-up of 30.6 months and a median of 26.9 months (6.5–93 months). Ninety five children had 24 months or more follow-up for their second hip. Forty six children were followed for their second hip between 12 and 24 months. The 15 children that were followed for their second hip less than 12 months all had a radiographically confirmed closure of the capital physis.

The boy who emigrated did so within 6 weeks after surgery for the index hip. Another boy died 22 months’ post index hip surgery.

The median age at surgery for the girls in this group was 11.2 (7.2–15.1) years and for the boys, it was 13.0 (9.5–17.7) years.

Among the 201 children treated without prophylactic surgery 43 (21%) later developed SCFE in the contralateral hip within the follow-up time of 24 months. For the group of 201 children treated without prophylactic surgery, the median age when diagnosed with SCFE in the index hip for the girls was 12.1 (8.4–14.7) years and for the boys it was 13.4 (3.8–16.7) years.

### Hospitals

When the study period started in 2007 there were 39 hospitals in Sweden that treated children with SCFE. During the study period, five hospitals changed their treatment protocol and referred children with SCFE for surgical treatment. Between 2007 and 2013 there were 20 hospitals that on average treated >1 child with SCFE per year, four of these on average treated >2 children per year, and three treated on average > 3 children per year.

## Discussion

To our knowledge, this is the first prospective cohort study that describes the epidemiology for SCFE based on a total national population. It was possible to reach 100% completeness using the definition: the proportion of all children treated for SCFE that were registered in the population covered by the Swedish National Patient Register (NPR). The Swedish NPR database does not allow for the assessment of bilateral disease and cannot discriminate between first-time admission for the treatment of SCFE versus readmission for complications, hardware removal, or contralateral disease.

A thorough retrospective comparison for personal identity numbers registered in NPR with surgery performed for SCFE and the corresponding medical records was made where we found 35 children that were initially not reported but that could be retrospectively included after informed consent was obtained.

The median age at diagnosis was similar to that reported previously from Southern Sweden 1960–1969 [28] and Gothenburg, Sweden, 1946–1992 [12]. During the twentieth century, a gradual decrease in age at onset of SCFE was seen, but this trend has now stopped [57, 58].

In this study, the male-to-female ratio was 1.3:1. Previous reports have shown a ratio with variations from 1.1:1 up to 4.1:1 [1, 2, 23, 25, 28, 33, 34], but the difference between boys and girls has gradually levelled out with time.

We managed to obtain figures for 81% of the cohort population concerning age-adjusted Body Mass Index (BMI). For the boys 76% had overweight or were obese whereas for the girls this was only true for 56%. In a recent population-based study in Sweden it was found that 17% of boys and 18% of girls 15 years old had overweight or were obese [59]. The increase in incidence for SCFE has been proposed in Scotland to be linked to an increase in BMI among children [60]. We had 43% girls in this population-based study for SCFE of the index hip and we believe that overweight or obesity should still be considered as a major characteristic for boys but not necessarily for girls. This will be important information to distribute to all professions who primarily attend children with hip or knee pain.

The average annual incidence calculated in this study was for children that were 9–15 years old in Sweden during the period 2007–2013. It is difficult to compare reports of incidence of SCFE because the disorder is related to ethnicity [1]. Other authors have used different methods to present the incidence rate: (1) attack rate calculated as the sum of annual incidence rate for every age group (7–17 years) over the age group of risk [12], (2) incidence rates for children aged ≥9 to ≤16 with combined data for 2 years [2], (3) overall incidence for children aged 7–18 years during a 12-year period in relation to the total number of children of those age groups during 1 year in the middle of the study period [61], (4) the total number of surgical procedures for SCFE over 13 years for children between 5 and 19 years related to the average number of boys and girls in that age cohort during the study period [23], (5) annual age-specific incidence rates calculated as the number of new cases of SCFE per 100,000 paediatric population (aged 9–16 years, inclusive) [22] or (6) incidence of the number of patients born in the same year related to the number of births during that year [28]. Despite the various methods used to calculate incidence, if we compare our data with the incidence previously reported from Southern Sweden [28], 6.1/10000 living born for boys and 3.0/10000 living born for girls, there seems to be an increase in incidence for girls, but not for boys. We have no explanation for this.

The severity of the slip was correlated with the duration of symptoms [14–18]. The 44 children with knee pain as presenting symptom had a 2 months longer median duration of symptoms until diagnosis compared with children having initial hip/groin pain. The children with knee pain also had a lower rate of referral for a radiographic hip examination at the initial health care contact than the children with hip/groin pain [62].

We used the clinical classification according to Loder [49] for the stability of the capital physis because this did not require any new preoperative diagnostic methods to be implemented. When this study was initiated in 2007 this classification was widely accepted [37, 42, 63–67] but there are reports that argue against this classification because even within this group the degree of stability of the physis has been shown to vary [41, 68–72].

There was a minor peak of incidence during our summer (Jun-Aug) and the lowest incidence was found in the winter (Jan-Mar) but the explanation for this remains uncertain [27–30].

Concerning the co-morbidity issue, the most common diagnosis in this study was neuropsychiatric disorders (15 of 379). These children might have a different risk behaviour in recreational activities that could contribute to this situation [73]. All children below 2 years in Sweden are routinely offered supplementary Vitamin D and parents are encouraged to allow their children to be exposed to the sun that might explain our very low number of co-existing Vitamin D deficiency in this cohort.

Most children were treated with a percutaneous method using an implant that will allow further growth of the femoral neck [74]. Only 11 capital realignment procedures were performed in Sweden during 2007–2013 as primary treatment for SCFE in the index hip. There are different treatment protocols for unstable SCFE where an intentional closed reduction manoeuvre was used for 31/55 children, but joint aspiration of the hip in an unstable SCFE with the attempt to further reduce the intracapsular pressure [43, 44, 75] was only used for 5/55.

In the present study, 43% of the contralateral hips had a prophylactic fixation performed. The rationale for a programme where prophylactic fixation is always performed is controversial [76–84] except for children with metabolic or endocrine disorders [6, 9] and for the very young [85, 86]. Most hospitals in Sweden where prophylactic fixation is not routinely performed have a follow-up programme with repeat radiographic examinations of both hips until physeal closure of the proximal femur has occurred. The girls who received prophylactic surgery on the contralateral hip were younger (median age 11.2 years) than girls who were scheduled for regular radiographic follow-up (median age 12.1 years). We found no such difference for the boys. Menarche might be used in some hospitals as a cut-off point for the girls after which routine prophylactic fixation is not performed, whereas for the boys there is no such clear pubertal start point.

Loder et al. [87] reported that 80–90% of later SCFE in the contralateral hip developed within 18 months. We chose to follow up 24 months after the index hip was treated. Studies with follow-up into adulthood report the incidence of bilaterality to be as high as 63% [23, 24, 76, 77, 81, 87–94]. We advocate that all children, who have not undergone prophylactic surgery, should be scheduled for regular radiographic follow-up until closure of the capital physis.

The number of hospitals in Sweden treating SCFE has decreased from 39 to 34 since January 2007. Only three hospitals treat on average > 3 children per year for SCFE in the index hip. Sweden has areas that are not so densely populated and a referral to an orthopaedic centre with more experience of surgical treatment in paediatric orthopaedics might not be appropriate for all children with a stable mild or moderate SCFE. Continuous education will be of utmost importance for surgeons in hospitals that annually receive a low volume of children with SCFE. Probably children with a severe and/or unstable SCFE should be considered for a referral to a paediatric orthopaedic centre.

## Limitations

The children included in this study were followed for 24 months for any contralateral slip, but not all were followed until closure of the capital physis. A small number of hospitals followed their paediatric patients with regular radiographs routinely for only 12 months postoperatively, thus the true incidence of bilateral involvement might have been slightly underestimated.

In the analysis for the duration of symptoms in months, we were not able to separate the duration of patient’s delay versus doctor’s delay.

For the analysis of initial health care provider and the rate of referral for a diagnostic hip examination, we were not able to collect all medical records from outpatient visits prior to the primary surgery for SCFE at various health care providers. We used the history that was presented on hospital admission for primary surgery for SCFE.

Children in this cohort study might have had their surgery for SCFE in the index hip performed abroad during a vacation. They have therefore not automatically been included in this study. To our knowledge, only one child with a Swedish personal identity number during the study period was followed up in Sweden after such an event. There were no children treated for their second hip during the study period where the index hip had been treated before the family immigrated to Sweden. Families with children prone to develop SCFE might have emigrated during the study period, but only one child with SCFE in the index hip during the study period emigrated during the follow-up of 24 months for a contralateral slip.

## Conclusion

The average annual incidence of SCFE was 4.4 per 10,000 girls and 5.7 per 10,000 boys 9–15 years old. Most children were treated with percutaneous pin or screw fixation with an implant that would allow further growth of the femoral neck. Prophylactic fixation of the contralateral hip was performed in 43% of the children. Overweight or obesity is one major characteristic for boys with SCFE but to a less extent for girls. Knee pain as initial symptom cause a delay in diagnosis.

We recommend that all hospitals, where prophylactic surgery is not advocated for the contralateral hip, should implement a programme that includes regular radiographic follow-up until the closure of the proximal femoral physis has been verified. The number of hospitals that perform surgery for SCFE has been reduced, but still most of the hospitals in Sweden treat less than two children per year.

## Abbreviations

AP: Anteroposterior
